# Modeling heterothermic fitness landscapes in a marsupial hibernator using changes in body composition

**DOI:** 10.1007/s00442-023-05452-4

**Published:** 2023-10-05

**Authors:** Tamara Abarzúa, Isidora Camus, Felipe Ortiz, Abel Ñunque, Francisco A. Cubillos, Pablo Sabat, Roberto F. Nespolo

**Affiliations:** 1 Instituto de Ciencias Ambientales y Evolutivas, Universidad Austral de Chile, Valdivia, Chile; 2 Departamento de Biología y Química, Universidad de Santiago de Chile, Santiago, Chile; 3 Millenium Nucleus of Patagonian Limit of Life (LiLi), Valdivia, Chile; 4 Millennium Institute for Integrative Biology (iBio), Santiago, Chile; 5 Facultad de Ciencias, Universidad de Chile, Santiago, Chile; 6 Center for Applied Ecology and Sustainability (CAPES), Departamento de Ecología Facultad de Ciencias Biológicas, Pontificia Universidad Católica de Chile, Santiago, Chile

**Keywords:** Correlational selection, Selection surface, *Dromiciops*, Hibernation, Quantitative magnetic resonance, Isometric scaling

## Abstract

Hibernation is an adaptive strategy that allows animals to enter a hypometabolic state, conserving energy and enhancing their fitness by surviving harsh environmental conditions. However, addressing the adaptive value of hibernation, at the individual level and in natural populations, has been challenging. Here, we applied a non-invasive technique, body composition analysis by quantitative magnetic resonance (qMR), to calculate energy savings by hibernation in a population of hibernating marsupials (*Dromiciops gliroides*). Using outdoor enclosures installed in a temperate rainforest, and measuring qMR periodically, we determined the amount of fat and lean mass consumed during a whole hibernation cycle. With this information, we estimated the daily energy expenditure of hibernation (DEE_H_) at the individual level and related to previous fat accumulation. Using model selection approaches and phenotypic selection analysis, we calculated linear (directional, *β*), quadratic (stabilizing or disruptive, *γ*) and correlational (*ρ*) coefficients for DEE_H_ and fat accumulation. We found significant, negative directional selection for DEE_H_ (*β*_DEEH_ = − 0.58 ± 0.09), a positive value for fat accumulation (*β*_FAT_ = 0.34 ± 0.07), and positive correlational selection between both traits (*ρ*_DEEH × FAT_ = 0.24 ± 0.07). Then, individuals maximizing previous fat accumulation and minimizing DEE_H_ were promoted by selection, which is visualized by a bi-variate selection surface estimated by generalized additive models. At the comparative level, results fall within the isometric allometry known for hibernation metabolic rate in mammals. Thus, by a combination of a non-invasive technique for body composition analysis and semi-natural enclosures, we were characterized the heterothermic fitness landscape in a semi-natural population of hibernators.

## Introduction

Energy in ecosystems is limited, and animals obtain it via foraging, digestion, and absorption, and allocate it in parallel to biological functions such as growth, reproduction, and maintenance (Bochdansky et al. 2005; Brown et al. 1993; Weiner 1987). Then, ultimately, natural selection promotes the schedule that maximizes energy transfer from food to offspring, given environmental constraints (Roff 2002). This could be reflected in directional and positive selection gradients (Arnold 1983), if traits represent exceptional capacities enhancing survival and reproduction (e.g., aerobic capacity in endotherms, see Boratynski and Koteja 2009, 2010; Hayes and O'Connor 1999; Jackson et al. 2001; Sadowska et al. 2015). On the opposite side of this spectrum, a positive balance in energy budget could be attained by minimizing maintenance costs; “austere” phenotypes (Artacho and Nespolo 2009b; Bochdansky et al. 2005; Boratynski 2020; Boratynski et al. 2020; Mueller and Diamond 2001; Schimpf et al. 2012). Mammalian folivores for instance (e.g., pandas, sloths, and koalas), are examples of austere strategies to life, due to the constraints of processing a low-energy diet (Cork et al. 1983; Krockenberger and Hume 2007; Nie et al. 2015; Pauli et al. 2016). Tropical birds also exhibit a low pace-of-life strategy (Class and Moore 2013; Wiersma et al. 2007); and also populations of rodents exposed to low productivity during several generations (Mueller and Diamond 2001). In this context, the “logical” solution (sensu Schmidt-Nielsen 1995) is to reduce metabolism facultatively under unfavorable conditions, a capacity of several microorganisms, plants and animals, named with by a range of terms (e.g., diapause, aestivation, cold hardening, brumation, daily and seasonal torpor), and known collectively as “metabolic depression” (Guppy and Withers 1999).

Metabolic depressions are non-pathologic and reversible reductions in metabolic rate below the normal levels (Guppy and Withers 1999), allowing transient survival when conditions are incompatible with life. These conditions include cold, absence of food, and desiccation. Mammalian hibernation (“seasonal torpor” or “multiday torpor”, see definitions in Geiser and Ruf 1995) is a particularly well-known case of metabolic depression, which have been intensely studied since the late nineteenth century (Lyman 1948; Lyman et al. 1982; Pembrey and White 1896). During hibernation, animals (e.g., many bats, rodents, small marsupials, black and brown bears) remain in a state of “suspended animation” (= torpor episodes) for several days or weeks, sheltered in dens, caves, or tree cavities, avoiding predators, storms, fires, floods, heat waves, and droughts (Barak et al. 2018; Nowack et al. 2016, 2020). During torpor, animals (especially, fat-storing hibernators, see Giroud et al. 2021) do not ingest food and rely completely on their adipose tissues, and they typically undergo pre-hibernation fattening in autumn.

Despite the evident adaptive significance of hibernation and daily torpor (hereinafter referred to as the “heterothermic phenotype”), few authors have characterized this phenotype at the intra-population level. This is important, because to respond to natural selection a trait should exhibit: (1) consistent variation (i.e., significant repeatability; Boratynski et al. 2019; Dohm 2002; Hayes et al. 1998; Labocha et al. 2004) and (2) fitness consequences (i.e., a significant selection gradient; Arnold 1983; Lande and Arnold 1983). Several authors have addressed these questions in a range of organisms, populations, and traits in what is known collectively as “phenotypic selection studies” (Kingsolver et al. 2001; see reviews in Hoekstra et al. 2001; Kruuk et al. 2008; Mousseau and Roff 1987). However, few explorations of selection surfaces exists (i.e., bi-variate fitness profiles, according to Phillips and Arnold 1989) on the heterothermic phenotype (Boratynski et al. 2019; Dammhahn et al. 2017; Lane et al. 2011). Some authors used heterothermic indexes, quantifying the extent of heterothermy based on body temperatures, *T*_B_s (Boyles et al. 2011), which resulted in being practical for comparing large datasets on intra- and inter-specific heterothermic patterns (Boyles et al. 2013; Dammhahn et al. 2017). Interestingly, using this index, Dammhahn et al. (2017) reported consistent variation in heterothermic traits in free-ranging eastern chipmunks (*Tamias striatus*), suggesting that fluctuating selection maintains the heterothermic polymorphism (Dammhahn et al. 2017). These authors also found that the heterothermic index is repeatable, thus exhibiting consistent inter-individual variation (see also Boratynski et al. 2019). On the other hand, Lane et al. (2011) estimated significant heritabilities for another aspect of hibernation (emergence day, in Columbian ground squirrels), which represents potential to respond to selection (Lynch and Walsh 1998).

Although heterothermy descriptors such as heterothermy indexes or emergence date provide valuable information about the frequency or duration of torpor and hibernation, these do not necessarily quantify the amount of energy that is actually saved during hibernation (see a discussion in Geiser 2020). In this study, we analyzed a population of an opportunistic hibernator (sensu Bozinovic et al. 2004), the microbiotheriid marsupial monito del monte (*Dromiciops gliroides*). This is a fat-storing hibernator which is known to exhibit short bouts of torpor and also seasonal, multiday torpor (Bozinovic et al. 2004; Nespolo et al. 2021; Nespolo et al. 2010). We followed the hibernation cycle, under semi-natural conditions, assuming that winter is a selective event in which survival differentially selects for individuals maximizing energy acquisition and/or minimizing expenditure. In some species, mortality in winter can reach 70% (Bearman-Brown et al. 2020; Juskaitis 1999), which represents a selective event promoting survival in those individuals that optimize energy use. However, in some species, survival during hibernation is high, compared with other periods, such as the breeding period (Le Coeur et al. 2016), or it vary with external perturbations (Boyles and Brack 2009).

If winter represents a selective event, then we predict differential survival of those individuals maximizing previous fat accumulation, and/or minimizing energy expenditure during hibernation. However, these strategies could take several forms in terms of magnitudes and forms of selection. It could be directional, negative if selection promotes the reduction in energy expenditure (Artacho and Nespolo 2009a), or it could be sex specific if some sex is more energetically constrained than the other (Boratynski et al. 2010). Selection could be correlational if joint effects on expenditure and other traits are involved (Bartheld et al. 2015) or positive and directional, if energy expenditure is correlated with other capacities enhancing survival (e.g., resistance to pathogens, see Guerreiro et al. 2012).

Fat-storing hibernators do not normally ingest food during hibernation (Boyles and Brack 2009), which facilitates the experimental analysis of energy accumulation and/or consumption, provided a method for analyzing in vivo body composition. In this study, we used quantitative magnetic resonance (qMR), a non-invasive procedure for quantifying body composition in small animals. Using this procedure, we estimated daily energy expenditure of hibernation (DEE_H_), fat accumulation and survival, and modeled a bi-variate selection surface for the heterothermic phenotype.

## Methods

### Field enclosures

We installed eight cylindric enclosures in the same location as for captures (San Martin Biological Station, 39° 38′ 50.71″ S, 73° 11′ 46.43″ O), which were distributed within the forest and separated about 5 m from each other, covering a total area of about 80 m^2^ (see Fig. 1 in Nespolo et al. 2022a). Each enclosure had an internal volume of 2 m^3^ and was manufactured in zinc with a large 1.8 m-diameter cylinder buried 10 cm in the ground, which gave a 0.8 m-height above ground. The ceiling was framed in timber and had a mesh that allowed the entrance of light and humidity, but avoided the animals' escape or predators’ attack. Then we included a tri-dimensional arrangement of *Nothofagus* twigs and logs, with native bamboo (*Chusquea quila*) within the enclosure. The floor was covered by mosses and bamboo leaves, which are essential for *D. gliroides* nest building (Hershkovitz 1999; Honorato et al. 2016). We also included two removable hibernacula per enclosure, and a temperature data-logger (HOBO^®^, Onset, USA) for continuous recording of air temperature. Water was provided ad libitum in a plastic plate (1L). The enclosures were cleaned weekly, to prevent any invertebrate from entering by any means.

### Animals, treatments, and experimental hibernations

48 monitos (body mass, *M*_B_ = 35 g, se = 5 g) were captured in February 2022, during nighttime, using 100 tomahawk traps attached to trees 2 m above ground, and baited with banana. Animals were transported to the outdoor enclosures after capture, which were located 20 m away from the trapping site and maintained with ad libitum food and water. Food consisted in a mix of cat food pellets, banana and apple, which provides all needed nutrients to this omnivorous marsupial (Contreras et al. 2014; Nespolo et al. 2022a). Each captured individual was tagged using a PIT-tag subcutaneous chip (BTS-ID, Sweden) to allow subsequent identification. The sample was divided into two (24 and 24 individuals; 6 individuals per enclosure), to assign each half to a “periodic” and an “undisturbed” measurement of body composition using quantitative magnetic resonance (qMR, see below). This division into two treatments was applied because disturbing the animals in the middle of hibernation induces rewarming, which increases energy costs (Boyles and Brack 2009) and would overestimate DEE_H_. Given there is discussion regarding the exact energy cost of rewarming (Geiser 2007; Stone and Purvis 1992), we decided to include this treatment. Unfortunately, a windstorm knocked over a tree on one of the undisturbed treatment exclusions, and all individuals escaped leaving an unbalanced design (24 and 18 individuals for periodic and undisturbed treatments, respectively).

Experimental hibernation was initiated at the first qMR measurement, which was taken in April 13th (= day 0). On this day, all animals were moved to the laboratory and scanned by qMR. Also, food was removed 24 h before this first qMR measurement. Both groups were taken to the enclosures and released. The undisturbed group was measured again at the end of the experiment, which occurred at day 156 (Sept 19th). The periodic group was taken to the laboratory for qMR measurements, approximately every 40 days. The first qMR record was taken at day 0, then second at day 42, the third at day 77, the fourth at day 126, and the fifth at day 156 (end of hibernation). Given that DEE_H_ was computed between qMR records, we calculated four measures for the periodic treatment and only one for the undisturbed treatment. All animals were normothermic at the moment of qMR measure.

All animals entered torpor within the first 48 h of the experiment and remained torpid during the whole period. This was confirmed by two facts. *First*, enclosures were visited every 2 days to confirm that the animals remained torpid (cold after touched, unresponsible, clustered in small groups). Only 7 individuals, out of 48, after the whole experiment, were seen to be active during these visits. *Second*, the time series of *T*_B_ of animals with data-loggers showed the typical torpor pattern of hibernating species (i.e., torpor episodes of several days of duration, interspersed by short rewarming events, see next section). Importantly, previous experiments with this species have revealed the same pattern: animals do not interrupt hibernation unless experimentally treated with excess of food (e.g., Nespolo et al. 2022a). The trigger of torpor, which occurs usually in 100% of the individuals after 24–48 h of food withdrawal, and under cold conditions (*T*_A_ below 10 °C), has been confirmed several times during short experiments of food deprivation in semi-natural conditions (Mejias et al. 2022), by laboratory experiments (Cortes et al. 2014; Nespolo et al. 2010), or by time series of T_B_ recorded by data-loggers in the field (Nespolo et al. 2021), as shown here (see next section).

### Intraperitoneal data-loggers

We used five miniature data-loggers (Star-Oddi DST nano, 1.3 g, cylindric, 17 mm long, 6 mm in diameter) that were set to record body temperature (*T*_B_) every 5 min. The devices were surgically implanted into the abdomen (intraperitoneal) of two individuals of the undisturbed treatment and three individuals at the periodic treatment. This was performed 2 months before the experiment (April). According to the manufacturer, the devices were calibrated in a factory over a temperature range of 5–45 °C. Additionally, the data-loggers were calibrated by us in a beaker with water at 40 °C that was allowed to cool to room temperature (10 °C), with temperature records made every 2 min using a laboratory thermometer (alcohol). The linear regression between water and data-logger temperature (20 points) was highly significant (*R*^2^ = 0.99, *p* << 0.001). For both implantation and removal, we used subcutaneous tramadol 5 mg kg^−1^ and inhalation anesthesia for induction (isoflurane, 5%) and maintenance (isoflurane, 2.5%). We then administered subcutaneous meloxicam 0.5 g kg^−1^. The surgical approach consisted of a small incision (3 mm) on the abdominal region in their median plane, from the xiphoid process to the marsupium. The device was delicately placed perpendicularly to the body axis between the layers of the peritoneum, and the wound was closed with a stitch using sutures that are self-absorbing, both in the muscular plane and in the skin. The whole procedure lasted less than 5 min per animal. After this, the animal was maintained in the clinic for 5 d for recovery (under outdoor conditions), with food and water ad lib. The data-loggers were removed at the end of the experiment, in September.

### Quantitative magnetic resonance (qMR) and energy consumption

The qMR scanner we used was an EchoMRI 500 (Houston, Texas, USA), which has been validated several times in wild animals (Eastick et al. 2020; Kraft et al. 2019; Mejias et al. 2022; Riley et al. 2016). It gives instantaneous measures of body composition in less than one minute per animal. At each measurement, the animal was placed in an acrylic cylinder (5 cm diameter, 60 cm long) and immobilized by a Velcro-secured plunger to the cylinder. Then it was introduced into the magnetic resonance module, which was previously programmed for three scans. The whole process took 5 min per animal. Each time the coefficient of variation of these repetitions exceeded 6% (usually due to movement of the animal within the probe), the measurement was repeated. We calibrated the qMR scanner daily before every batch of measurements according to manufacturer’s recommendation, using a known sample of canola oil located in the antenna.

The daily amount of energy consumed during hibernation was calculated as DEE_H_ = [39.7kJ g^−1^ × (fat mass consumed, in grams) + 23.6 kJ g^−1^ × (lean mass consumed, in grams)]/time period between measurements, in days (Mejias et al. 2022; Nespolo et al. 2022b). This approximates the energy consumed per animal, assuming that everything else is maintained constant during hibernation (e.g., water turnover and proteins). The approach has been validated against actual measurements of body composition in torpid and active *Dromiciops* (Mejias et al. 2022). Given that animals were not allowed to ingest food during the experiment (the usual condition for hibernating *Dromiciops,* no food ingestion), all energy consumption could be calculated from changes in body composition. Previous estimates using this approach indicated that monitos consume 0.18 g of fat per day of hibernation (= 8.86 kJ day^−1^)(Mejias et al. 2022), during the first hibernation month. Thus, at this rate well-fed monito with 20 g of fat would last about 111 days in hibernation. However, it is known that as the winter progresses, energy consumption of hibernators is reduced (Cranford 1978; Jonasson and Willis 2012).

### Repeatability and selection coefficients

For estimating inter-individual variation of our focal traits (DEE_H_ and fat accumulation; “fat” hereinafter), we calculated repeatability from the multiple measurements in the periodic treatment, as the intraclass correlation coefficient, *τ* (Dohm 2002; Lessells and Boag 1987). It was calculated using the variance components module of Statistica, for which we estimated the variance component of individuals (*V*_ind_) and error (V_error_), and then repeatability was calculated as *τ* = *V*_ind_/(*V*_ind_ + *V*_error_) and expressed as a percentage. For instance, a significant *τ* value of 0.6 means that 60% of the variance in the trait is significantly explained by the inter-individual component of variation.

As a proxy of fitness, we considered two metrics. Survival as a binomial variable (1 = survived, 0 = not survived) and survival as the number of days an animal survived in the experiment. Every individual that reached 2.0 g of fat was considered near death and was removed from the experiment, to avoid unnecessary deaths. This criterion was decided based on previous observations in the laboratory, where animals could not be recovered from torpor when having less than 1.5 g of fat. Below this threshold, animals need to be treated by a veterinarian and fed with intravenous fluid to survive.

For analyzing the joint effects of fat accumulation and DEE_H_ on survival, as independent variables (traits), we considered fat accumulation (fat mass measured at day zero) and DEE_H_ calculated between day 0 and 42 (first period; before any removal) for the periodic treatment and the DEE_H_ calculated for the whole period. All traits were standardized to mean = 0 and SD = 1 (i.e., the average was subtracted from each data, and then it was divided by the standard deviation of the sample). We also included in the model potential covariates and “dummy” factors, such as lean mass, sex, enclosure, and body mass. We ran generalized linear models (GLM, R-package) on the data, for estimating linear (*β*), quadratic (*γ*), and correlational (*ρ*) selection coefficients on the model: survival ~ intercept + *β*_1_DEE_H_ + *γ*_1_DEE_H_^2^ + *β*_2_fat + *γ*_2_fat^2^ + *ρ*DEE_H_ × fat. We first ran full models including potential covariates and dummy variables and applied information criteria by Akaike (AIC, MuMIn package in R) (Burnham and Anderson 2002) for model selection. Following Stinchcombe et al. (2008), the quadratic coefficient overestimates the real effect by twofold, then we divided it by two.

Bi-variate fitness surfaces (Phillips and Arnold 1989) were approximated using generalized additive models with integrated smoothness estimation (GAM) in R's mgcv package (Wood 2011). GAM estimates the degree of smoothness of each term using quadratically penalized likelihoods and approximates them with penalized regression splines. This combination of non-linear regression and parameter modeling generates accurate fitness surfaces, even with small sample sizes (Morrissey and Sakrejda 2013). Also, the use of splines allows for flexibility in the model fit and avoided overfitting. All the analyses are reproduced by a single R-script, provided together with the data, available upon request.

All procedures presented in this study were approved by the Chilean Agriculture, and Livestock Bureau (SAG) permits No 4371/2019 and 3393/2019, and by the Bioethics Committee of the Universidad Austral de Chile, resolution 313/2018 annex 2019. No animal was harmed in these procedures. All surviving individuals were released at the site of capture in September, after the study.

## Results

### Descriptive statistics

**Fig. 1:**
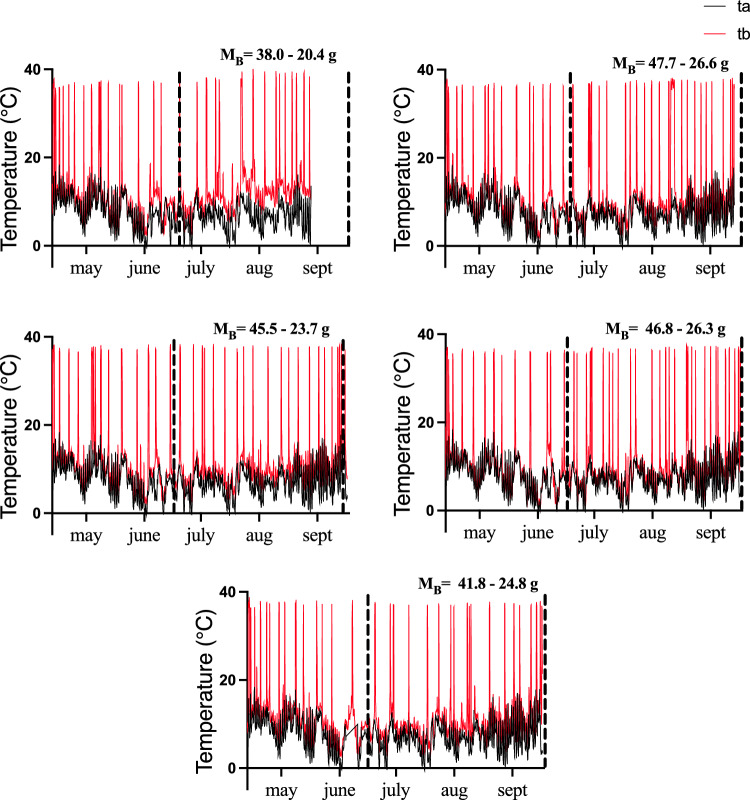
Time series of body temperatures (red, from intraperitoneal data-loggers) and ambient temperatures (black, from environmental data-loggers), in five individuals with intraperitoneal data-loggers. The change in body mass during the experiment is shown in each plot. Dotted lines represents the winter period. See text for details and methods

**Fig. 2:**
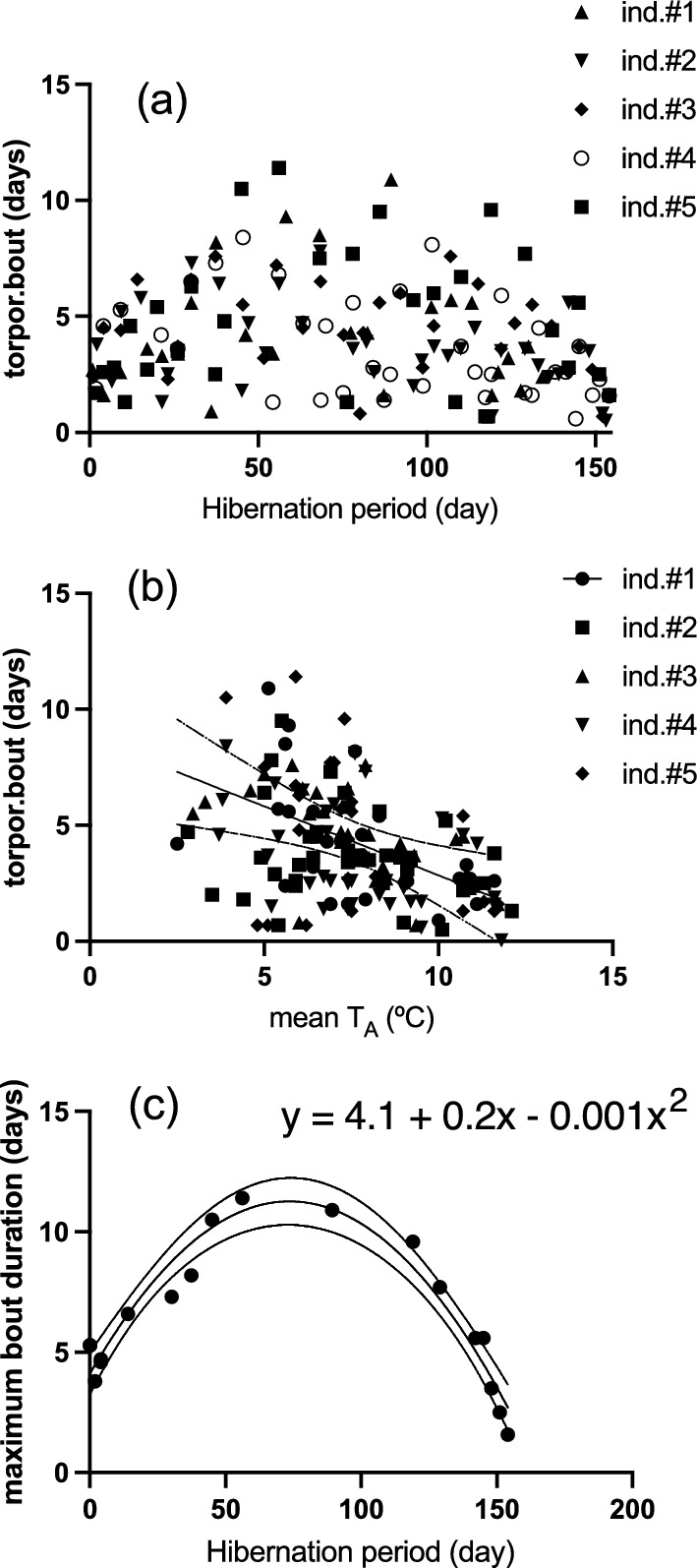
**a** Relationship between torpor bout duration and the hibernation period of the five individuals with data-loggers presented in Fig. 1. **b** Relationship between torpor bout duration and ambient temperature for the same individuals. **c** Maximum torpor bout duration, extracted from the time series of *T*_B_ (**a**), associated with the hibernation period. The dataset was adjusted to a parable (adjusted *R*^2^ = 0.90, *p* = 0.0001), for which the equation is indicated in the graph. Confidence interval of 95% was shown

All animals entered torpor within the first 48 h of the experiment and remained torpid during the whole period, experiencing periodic arousals as observed in the five individuals with data-loggers (Fig. 1). The pattern of torpor bout duration at different moments of hibernation is shown in Fig. 2a, which indicates a mean torpor bout duration of 4.1 ± 0.19 days (min = 0.04, max = 11.4 days). Also, we found a significant regression of torpor bout duration and *T*_A_ (slope = -0.6, intercept = 8.8 *R*^2^ = 0.27 *p* = 0.006, Fig. 2b). A non-linear quadratic adjustment of maximum torpor bout duration with hibernation period (i.e., the values at the edge of Fig. 2a, *n* = 18) provided to be highly significant (intercept = 4.11, linear term = 0.19, quadratic term = − 0.0013; *R*^2^ = 0.90; *p* = 0.001). This adjustment indicates that the mean torpor duration reached a maximum of 11.4 days at day 75, to be reduced gradually until the end of winter (day 150; Fig. 2c).

Initially, 24 individuals started the experiment in the “periodic” treatment, out of which 16 of them completed the whole experiment (= “survivors”, hereafter), and 8 individuals were removed due to death criterion (= fat mass below 2.0 g; “non-survivors”). These individuals did not wake up from torpor and had to be treated in the clinic with intraperitoneal fluid and heat, with which they managed to be rehydrated and finally came out of torpor. Three of them did not make it and died. After 3 weeks of feeding in the clinic, the other five regained their pre-hibernation weight and were released along with the rest.

**Fig. 3:**
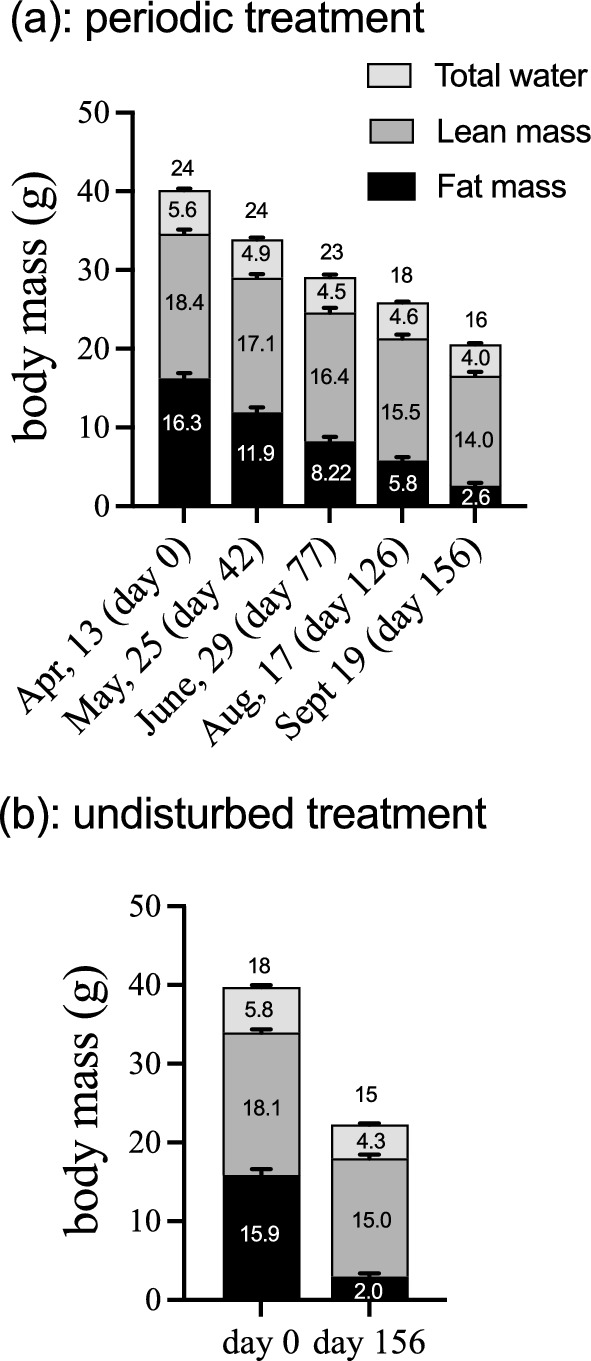
Changes in body mass and composition (total water, lean mass and fat mass, in grams; within bars) during the experimental period (means; error bars represent standard errors). **a** Represents the “periodic” treatment and **b** shows the “undisturbed” treatment (= only initial and final qMR measurements). Numbers above bars show sample size, and numbers within bars indicate means in grams

**Fig. 4:**
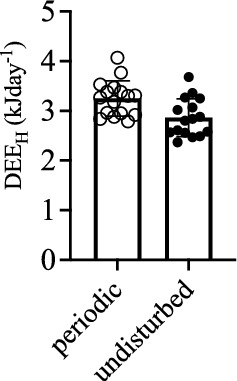
Daily energy expenditure of hibernation (DEE_H_) determined in individuals that were moved periodically to the laboratory for measurements of qMR and individuals that were measured only at the beginning and end of the experiment, showing a net increase of 0.39 kJ day^−1^ in periodically disturbed animals. Significant values are shown after an ANCOVA using lean mass as covariate (*F*_1,29_ = 6.2; *p* = 0.019)

On the other hand, from the set of 18 initial individuals in the “undisturbed” treatment, 15 of them reached the end of the experiment (only 3 removals, from which one died and the other two were recovered as indicated before). Also, water content and lean mass remained approximately constant, whereas fat mass was reduced from 16.3 ± 0.65 to 2.6 ± 0.37 g in the periodic treatment (Fig. 3a), and from 15.9 ± 0.74 to 3.0 ± 0.38 g in the undisturbed treatment (Fig. 3b). As expected, the reduction in fat and lean mass was reflected as a significant difference in a repeated measure comparison (before/after qMR), on both variables (fat: F_1,31_ = 1423.1; *p* << 0.0001, Fig. 3a; lean mass: *F*_1,31_ = 222.1; *p* << 0.001, repeated measures ANOVA, Fig. 3b). In the periodic treatment, animals consumed 77.1 ± 2.1% of fat and 22.9 ± 2.1% of lean mass and in the undisturbed treatment these numbers were 81.3 ± 1.3% (fat) and 18.7 ± 1.3% (lean). Lean mass consumption in the periodic treatment was significantly higher than in the undisturbed treatment (*t*_30_ = −2.4, *p* = 0.022). However, the comparison of DEE_H_ between periodic and undisturbed treatments (using initial and final sample) yielded significant, but small effects, revealing that animals in the periodic treatment spent, on average, 0.35 kJ day^−1^ (= 8.8%) more energy than undisturbed individuals (Fig. 4). We included this elevation as a fixed effect in the survival analysis.

### Inter-individual variation

**Fig. 5:**
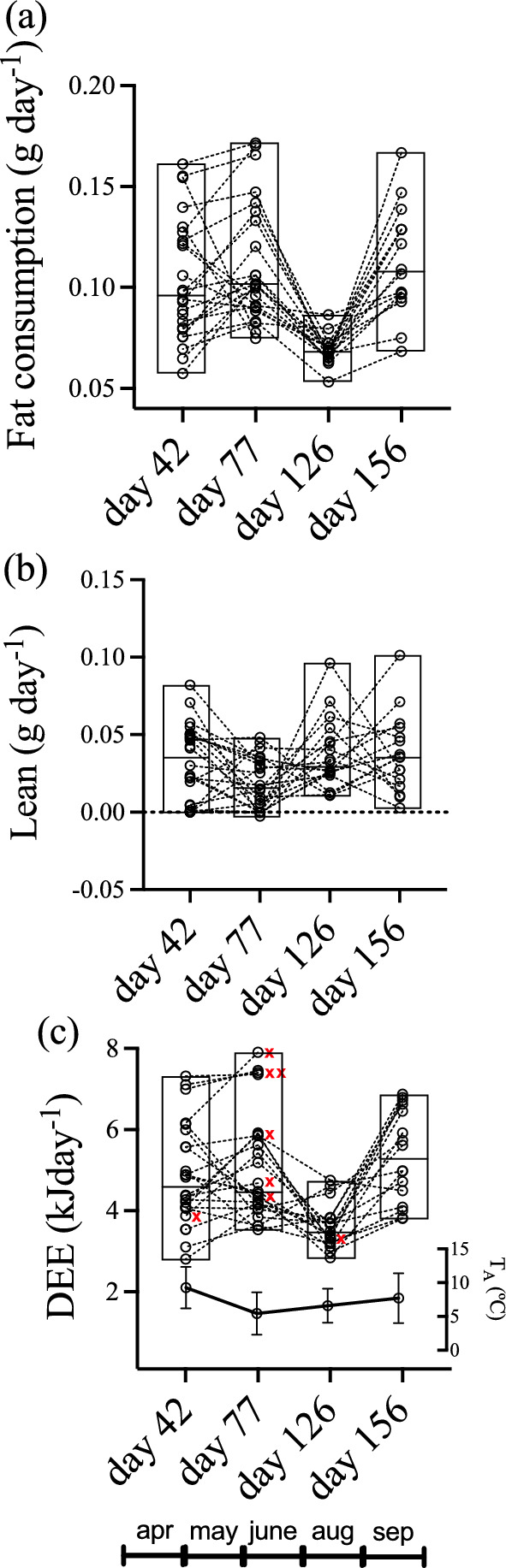
Time progression of fat (**a**), lean mass (**b,**) and energy consumption as DEE_H_ (**c**), expressed as individual reaction norms. Boxes represent medians and range. Red crosses show non-survivor animals. Mean ambient temperature at the experimental site is plotted as reference in **b**

The repeatability of body composition, that is, the time consistency of the trait in the sample, resulted high and significant only for lean mass (*τ* = 53.2%), but not for fat mass or body mass, which suggests that these traits do not maintain their ranking in the population (Table 1). However, DEE_H_ gave a significant repeatability (*τ* = 35.1%, see Table 1), thus suggesting that this trait does exhibit inter-individual consistent variation. To visualize the time progression of changes in body composition and DEE_H_, considering inter-individual variation and also identifying survivors and non-survivors in the sample, we plotted individual reaction norms (Fig. 5). These plot shows a shrinkage in fat consumption (Fig. 5a), resulting from a significant reduction in phenotypic variance to a third of the values, at day 126 (K-squared = 27.9, *df* = 3, *p* <  < 0.001, Bartlett test). This variance reduction is not observed for lean mass consumption (Fig. 5b; K-squared = 4.0, *df* = 3, *p* = 0.27, Bartlett test), but observed for DEE_H_ (Fig. 5c; K-squared = 12.7, *df* = 3, *p* = 0.005, Bartlett test). Consumption rates generated an overall DEE_H_ (periodic treatment) of 4.87 ± 0.26 kJ d^−1^, which was not homogeneous during hibernation. Significant differences were found among periods (*F*_3,45_ = 10.6; *p* < 0.00002, repeated measures ANOVA), which were generated by the low DEE_H_ of day 126 (= 3.82 ± 0.24 kJ day^−1^), compared with the other three periods (Fig. 5c).

**Table 1:** Repeatability analysis (intraclass correlation coefficient, *τ*, “tau”**)** for daily energy expenditure of hibernation (DEE_H_), lean mass, fat mass, and body mass of animals that were measured periodically

Trait	Variance component		*τ* (%)	*F*
	Individuals	Error		
DEE_H_	0.61	1.13	35.1	2.82***
Lean mass	3.52	3.10	53.2	4.82***
Fat mass	− 2.62	20.40	− 14.7	0.57 (n.s.)
Body mass	1.69	37.68	4.3	1.15 (n.s.)

*τ* = *V*_individuals_/(*V*_individuals_ + *V*_error_)

***p < 0.001

### Selection coefficients and selection surfaces

**Fig. 6:**
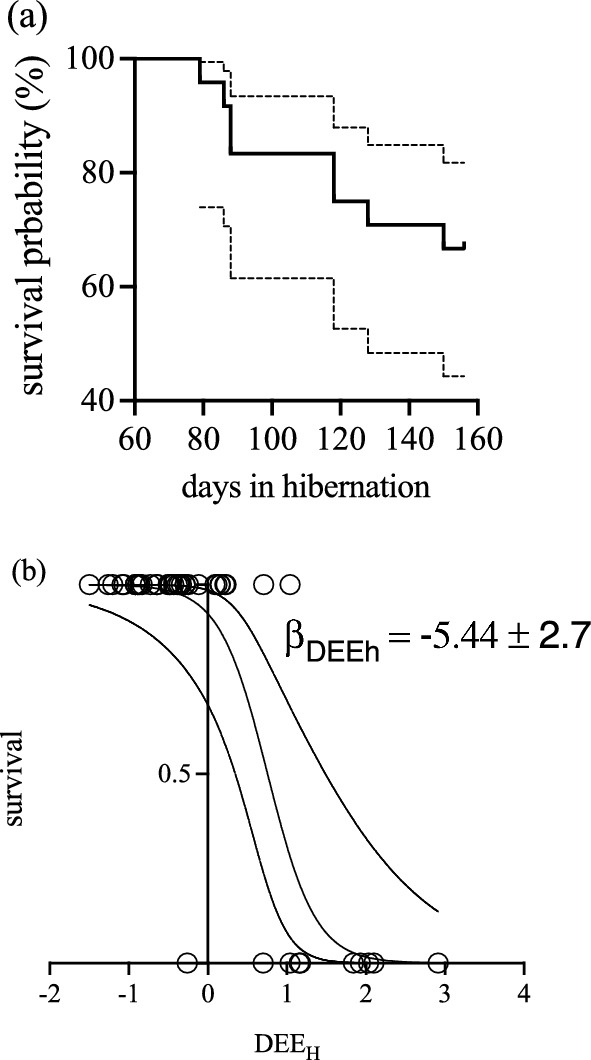
**a** Survival curve (± 95% confidence intervals, dotted line) of *D. gliroides* hibernation in our semi-natural enclosures. **b** Survival as binomial variable, in function of DEE_H_ (standardized traits to mean = 0 and SD = 1). The linear coefficient of selection is shown for DEE_H_, and was found to be significant (logistic regression with *p* = 0.045)

**Fig. 7:**
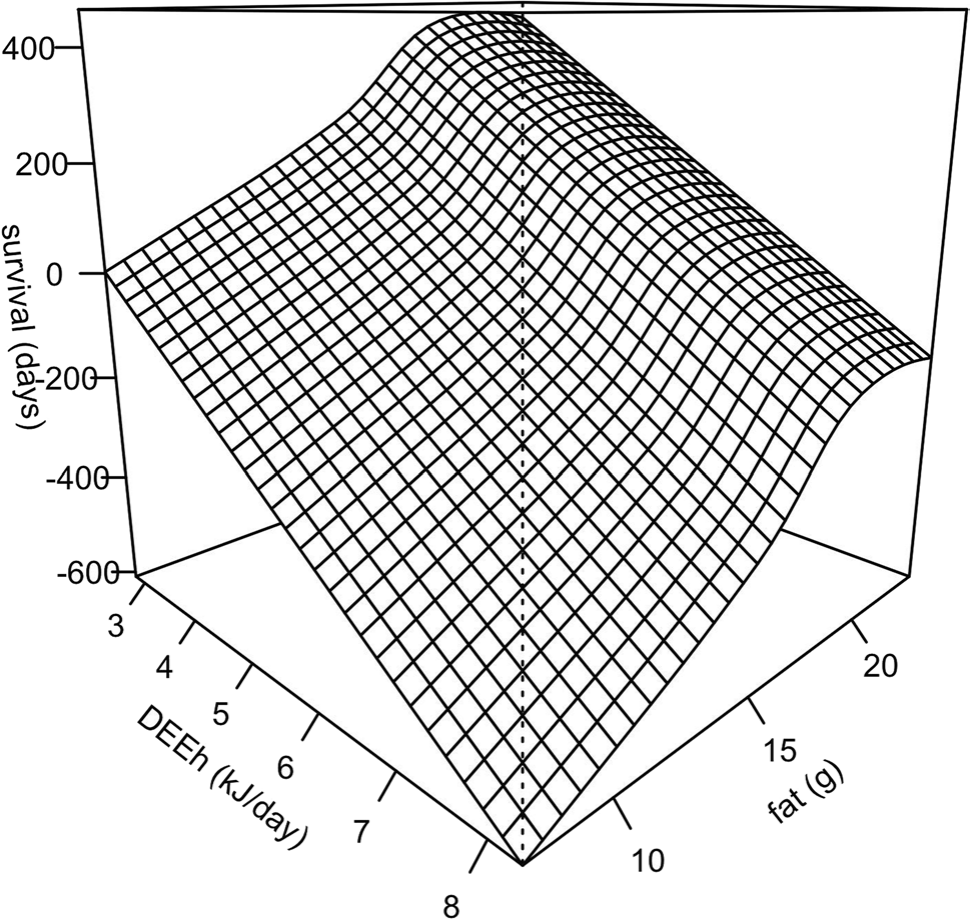
Correlational selection maximizing low DEE_H_ and high fat accumulation, represented by a fitness surface modeled using generalized additive models (adj-*R*^2^ = 0.82; deviance explained = 84%) and integrated smoothing parameters on the dataset using days of survival as fitness proxy. See “Results” and Table 4 for details

Non-survivors (denoted by a red cross in Fig. 5c) generated an overall survival of 70% (Fig. 6a). Survivors (*n* = 16) had a mean DEE_H_ of 4.35 ± 0.21 kJ day^−1^ which was significantly lower than for non-survivors (*n* = 8; 5.90 ± 0.48 kJ day^−1^; *t*_21_ = 4.6; *p* = 0.0001; *t* test). According to the AIC criteria, the best model explaining survival as a binomial variable was the fifth (AICc = 15.9, weight = 0.48, see Table 2), which considers only linear coefficients for both, DEE_H_ and fat. However, after testing these effects with the GLM, only DEE_H_ resulted with a significant linear coefficient (Fig. 6b: *β*_1_ = − 5.44 ± 2.7, *p* = 0.045). Also, the difference between the best model and the second in the ranking is below 2.0, thus they can be considered statistically equivalent. For the case of the continuous proxy of survival (days of survival), the best model according to the AIC criteria was the one considering all linear, quadratic, and correlational effects, but dropping “dummy” variables (Table 3, model 7). This GLM yielded significant effects for the linear coefficients in both traits, and also for the correlational coefficient (Table 4; *ρ* = 0.24 ± 0.07; *p* = 0.0012). A positive correlational coefficient indicates that extremely low values of one trait (here, DEE_H_), combined with extremely high values of the other trait (fat accumulation, in our data), maximizes survival. This can be visualized in the selection surface obtained by the GAM adjustment (Fig. 7).

**Table 2:** Ranking of the best model according to AIC criteria (smaller is better), using survival as a binomial response

Model	Expression	logLik	AICc	dAICc	Weight
5	Survival ~ DEE_H_ + FAT	− 4.64	15.9	0	0.48
2	Survival ~ treatment + DEE_H_ + FAT + 0.5 × DEEH^2^ + 0.5 × FAT^2^ + DEE_H_ × FAT	0	17.1	1.31	0.25
4	Survival ~ treatment + DEE_H_ + FAT	− 4.58	18.2	2.31	0.25
3	Survival ~ treatment + DEE_H_ + FAT + DEE_H_ × FAT	− 3.91	19.4	3.54	0.081
6	Survival ~ DEE_H_	− 8.09	20.5	4.59	0.048
1	Survival ~ SEX + ENCLOSURE + BODY MASS + LEAN MASS + treatment + DEE_H_ + FAT + 0.5 × DEE_H_^2^ + 0.5 × FAT^2^ + DEE_H_ × FAT	0	30.5	14.62	0

*DEE*_*H*_ daily energy expenditure of hibernation, *FAT* fat accumulated prior to hibernation, *treatment* periodic or undisturbed

**Table 3:** Ranking of the best model according to AIC criteria (smaller is better), using survival as a continuous response (days of survival)

Model	Expression	logLik	AICc	dAICc	*w*
7	Survival ~ DEE_H_ + FAT + 0.5 × DEEH^2^ + 0.5 × FAT^2^ + DEE_H_ × FAT	− 18.9	55.0	0.0	0.47
3	Survival ~ treatment + DEE_H_ + FAT + DEE_H_ × FAT	− 20.7	55.7	0.75	0.32
2	Survival ~ treatment + DEE_H_ + FAT + 0.5 × DEEH^2^ + DEE_H_ × FAT	− 18.4	57.1	2.15	0.16
5	Survival ~ DEE_H_ + FAT	− 25.7	60.5	5.5	0.030
4	Survival ~ treatment + DEE_H_ + FAT + DEE_H_ × FAT	− 25.6	62.8	7.9	0.009
1	Survival ~ SEX + ENCLOSURE + BODY MASS + LEAN MASS + treatment + DEE_H_ + FAT + 0.5 × DEE_H_^2^ + 0.5 × FAT^2^ + DEE_H_ × FAT	− 15.2	64.8	9.8	0.004
6	Survival ~ DEE_H_	− 41.6	89.8	34.8	0.0001

*DEE*_*H*_ daily energy expenditure of hibernation, *FAT* fat accumulated prior to hibernation, *treatment* periodic or undisturbed

**Table 4:** Summary of a generalized linear model analysis on standardized variables, for estimating selection parameters of hibernation traits, according to the best model (model 7): survival (days) = intercept + *β*_1_DEE_H_ + *γ*_1_DEE_H_^2^ + *β*_2_fat + *γ*_2_fat^2^ + *ρ*DEE_H_ × fat

Coefficient	Estimate s.e		*t* value	*p* value
Intercept	0.13	0.10	1.31	0.019
DEE_H_ (*β*_1_)	− 0.58	0.09	− 6.08	< 0.0001
DEE_H_^2^ (*γ*_1_)	− 0.22	0.12	− 1.80	0.08
Fat (*β*_2_)	0.34	0.07	4.76	< 0.00001
Fat^2^ (*γ*_2_)	− 0.18	0.09	− 0.70	0.49
DEE_H_ × fat (*ρ*)	0.24	0.07	3.50	0.0012

*β* = linear selection coefficient; *γ* = quadratic selection coefficient; *ρ* = correlational selection coefficient

## Discussion

Heterothermy, including daily torpor and hibernation, is a generalized strategy that birds and mammals use to cope with cold, seasonal, and unpredictable environments. Extensive research over the past 40 years has documented the many ecological, mechanistic, and evolutionary aspects of the heterothermic phenotype (Boyles et al. 2020; Guppy and Withers 1999; Heldmaier et al. 2004; Lopez-Alfaro et al. 2013; Nespolo et al. 2022b; Ruf and Geiser 2015). Still, pressing questions such as if winter is a selective event, or whether fat reserves always last for the whole hibernation cycle, or whether there is inter-individual variation in the rate of energy depletion, are open research questions in the field. Here, we addressed some of these questions using a South American hibernator as a model organism. Our results indicate negative directional selection on DEE_H_ (if we consider survival as a binomial variable) and directional selection combined with correlational selection on DEE_H_ and fat accumulation (if we consider days of survival as proxy). Thus, for this model species, winter (and hibernation) represents a selective event. Also, we found that DEE_H_ is a repeatable trait, which suggests inter-individual consistent variation in this variable. In the following paragraphs, we discuss our results in terms of: (1) torpor bout duration, (2) periodic versus undisturbed effects, (3) the comparative context (4), selection surfaces in physiological traits, (5) the effects of warming on hibernators, and finally (6), caveats and prospects.

### Predicting torpor bout duration

With the records of body temperature, we were able to describe a negative pattern of torpor bout duration in function ambient temperature, as well as a non-linear pattern of torpor duration in function of date (see Fig. 2c). The maximum bout duration (days) is described by the expression (adj-*R*^2^ = 0.90): [maximum bout duration] = 4.1 + 0.2[hibernation period]−0.0013[hibernation period]^2^, which indicates that the maximum torpor duration is experienced at day 75 of hibernation (June, 25th), with torpor bouts of 11.8 days, to be reduced at day 150 (September, 13th), with bout durations of 4.9 days. When evaluated at y = zero (i.e., absence of torpor periods), the equation predicts that hibernation would last until day = 171. Of course, this does not consider fat contents, which could be depleted before this date. Although this pattern of torpor bout duration in hibernators is not new, for *Dromiciops* (Nespolo et al. 2021) or for other hibernators such as the little brown bat (Jonasson and Willis 2012), the jumping mouse (Cranford 1978), and the pigmy possum (Geiser and Ruf 2023), we think our results are interesting because they provide a predictive equation to be compared with other populations or species.

### Periodic versus undisturbed treatment.

We included a fixed factor to account for the possible metabolic elevation that manipulation represents, due to the forced arousals induced during those events, which in turn could have affected our survival estimations. However, we found this effect small (~ 9% of the total expenditure) and not contributing statistically to the final selection analysis. Still, this is a good opportunity to compare theoretical expectations with empirical values. The cost of every arousal from torpor for *D. gliroides* was calculated by Mejias et al. (2022) and does not surpass 4 kJ bout^−1^ (Table 2 in Mejias et al. 2022). Thus, assuming that every manipulation event induces one rewarming event, followed by an euthermic period of 5 h (the period of measurement plus transport), this would represent a metabolic elevation due to manipulation of about 38.3 kJ per event (assuming an energy expenditure in euthermia, in active animals, of 88  kJ day^−1^ see Nespolo et al. 2022a). The animals of the periodic treatment experienced three more disturbances than the ones in the undisturbed treatment, which means 114.9 extra kilojoules in a period of 156 days. This gives a theoretical difference of 0.74 kJ day^−1^ between treatments, which is about twofold the empirically obtained value in this study (= 0.35 kJ day^−1^, see Fig. 4). Thus, it seems that animals, in some way, compensated for those disturbances. Interestingly, the potential effect of disturbances (and subsequent arousals) on winter survival was modeled by Boyles and Brack (2009) for the small hibernating bat *Myotis lucifugus.* These authors assumed that every human disturbance is equivalent to 1 h of visit to the hibernaculum, producing one arousal event, and found that survival rates are not lowered substantially by a limited number of disturbances, as those arousals would have occurred naturally (Boyles and Brack 2009). Thus, it seems that animals, to some extent, adapt to disturbances perhaps varying periodic arousals and adjusting torpor deepness, as we see in *Dromiciops*.

### Energy savings of D. gliroides: comparative context

**Fig. 8:**
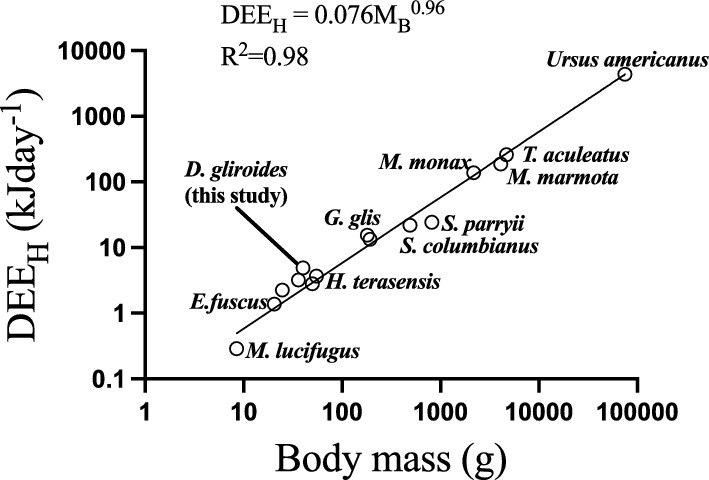
Comparative analysis of daily energy expenditure of hibernation (DEE_H_) using data from literature and our results for *D. gliroides* (modified from Nespolo et al. 2022b). Body composition during hibernation was recorded as body mass changes during hibernation for each hibernator

For calculating energy saved by hibernation and to put our results in a comparative context, we used the DEE_H_ of 4.87 kJ day^−1^ and compared it with literature (modified from Nespolo et al. 2022b), which produced a scaling relationship (Fig. 8). As observed previously (Heldmaier et al. 2004; Nespolo et al. 2022b; Ruf and Geiser 2015), the scaling of hibernation metabolic rate is isometric with mass, and that of *D. gliroides* falls slightly above the regression line (Fig. 8). Extreme hibernators of high latitudes, such as *Myotis lucifugus* (Humphries et al. 2002), or the arctic ground squirrel (*Spermophilus parryii*)(Barnes 1989), expectably, fall below the expected value in this allometry (Fig. 8). In terms of the allometric expectation, the observed DEE_H_ of *D. gliroides* is rather high, 186% of the expected value by size (Fig. 8), which is reasonable for a species from temperate climates. It would be interesting, however, to estimate DEE_H_ in high altitudinal populations of *Dromiciops*, where winter temperatures are well below zero (Mejias et al. 2021).

In a recent review, Wells et al. (2022) presented the distribution of hibernating species worldwide, showing not a single species in South America (see Fig. 4 in Wells et al. 2022). Thus, this field study and this short comparative analysis would justify the official inclusion of *D. gliroides* into the list of South American hibernators.

### Significant repeatability of DEEH

The fact that the repeatability of a trait is high and significant indicates that the trait maintains its ranking in the population and also suggests that there could be genetic variation associated with it (Boratynski et al. 2019; Bushuev et al. 2010). This is also interpreted as a potential to respond to natural selection (i.e., evolutionary potential, see Hayes 2010; Mousseau and Roff 1987). Then, it was interesting to find that both lean mass and DEE_H_ showed significant repeatability, but fat accumulation (and body mass) did not. In other words, survival to hibernation is mainly given by minimizing energy consumption rate, rather than by the maximization of energy accumulation capacity. This is consistent with previous studies analyzing repeatabilities of energy metabolism in wild mammalian populations (Boratynski et al. 2019; Labocha et al. 2004; Larivee et al. 2010; Nespolo and Franco 2007). However, it is well known that the capacity of fat accumulation is enhanced prior to hibernation in several species (Hogan et al. 2022; Kokurewicz and Speakman 2006; Kortner and Heldmaier 1995) and has been particularly well studied in the Alpine marmot, which maximizes energy assimilation by selective feeding and enhancing gastrointestinal capacity (Ruf et al. 2023). Thus, our results suggesting absence of inter-individual variation in fat accumulation capacity would be in contrast to this evidence and deserve further examination.

### Selection surfaces and physiological traits revisited

While we are not aware of previous studies analyzing fitness surfaces for visualizing the patterns of energy budgeting in hibernators, several authors have used these techniques before for analyzing trait–fitness relationships for morphological, physiological, and phenological traits of plants and animals (reviewed in Kingsolver et al. 2001; Svensson and Calsbeek 2012). For instance, Svensson and Sinervo (2000) analyzed the different shapes of fitness surfaces for egg mass and hatch day in a lizard, to conclude that competition is an important factor shaping selection pressures in the population. Also, Bartheld et al. (2015) used fitness landscapes for visualizing correlational selection on metabolic and body mass in a terrestrial invertebrate. Similarly, Pettersen et al. (2016) represented correlational selection using fitness surfaces to show that bryozoans with high metabolic rates in one stage and lower metabolic rates other are promoted by selection. In our case, the survival function suggests that individuals minimizing energy expenditure during hibernation and maximizing previous fat accumulation have a net benefit in survival. This result is consistent with the idea of austere phenotypes (Artacho and Nespolo 2009b), or the more general idea of the pace-of-life syndrome, in which animals that minimize energy turnover maximize survival and longevity (Careau et al. 2010; Wiersma et al. 2007), for which hibernators are a central example (Geiser and Turbill 2009; Turbill et al. 2011). However, the minimization of energy consumption with hibernation, at least for the case of *Dromiciops*, works well in the cold (however, see an exception in a marsupial in Geiser and Ruf 2023). Under conditions of warming, *Dromiciops* abort hibernation and remain active during winter, searching for food (Nespolo et al. 2022a, b). Under these circumstances, selection would promote lean and active, non-hibernating animals; but the rate of ongoing warming might be too fast for adaptations to keep up stable populations. While some hibernators could migrate and experience range shifts to colder regions (Humphries et al. 2002), the fate of a small marsupial attached to the temperate rainforest is uncertain (see below).

### The threats of global warming on heterothermic animals

*Dromiciops*, with its two described species (*D. gliroides* and *D. bozinovici*)(Quintero-Galvis et al. 2021), is known to be one of the few true South American hibernators, and the genus is also the sole living representative of a relict mammalian order (Microbiotheria), also recognized as the ancestral group of Australidelphia (Australian marsupials, see Feng et al. 2022). The high population densities described for *Dromiciops* (over 20 individuals per hectare, see Fonturbel et al. 2022) and the broad geographic range (over 1000 km, latitudinally, and at both side of the Andes, see Quintero-Galvis et al. 2021) are puzzling features for a relict species. Monitos can have a maximum litter size of only four pups and a single breeding event per year, a breeding period that is extremely long for a small mammal (over 3 months, see Fonturbel et al. 2022; Muñoz-Pedreros et al. 2005). The only possible explanation for the ecological success of *Dromiciops* is cold adaptation via heterothermy, the capacity to minimize energy expenditure by torpor episodes when food is unavailable. This efficient use of energy, combined with great foraging capacity during the active season, and efficient conversion capacity of food into tissues explain *Dromiciops* ecological success so far (reviewed in Fonturbel et al. 2022). However, an increase in only 1–2 °C in winter temperatures triggers arousal in this species (Nespolo et al. 2021), causing at least an 18-fold increase in food requirements (= from 4.87 to 88  kJ day^−1^; the active daily energy expenditure in winter animals, obtained from isotopic methods in Nespolo et al. 2022a). In this scenario, global warming could cause devastating consequences on hibernators. Indeed, it is known that the increases in winter temperatures for the region (subtropical Andes) for the next 20 years are projected to be in the order of 2.5 °C (IPCC 2019; Reboita et al. 2014).

### Merits, caveats, and limitations of this study

Summing up, our study provides significant insights into the physiology and ecology of a South American hibernator, by using a relatively novel, non-invasive technique (qMR) which allows precise estimations of body composition. This study has the merit, we believe, of addressing a question in physiological ecology (hibernation energetics) using evolutionary ecology approaches (fitness surfaces), a combination of tools not used very often. It also provides a detailed characterization of hibernation energetics, which will permit to parameterize predictive models of population stability under global change scenarios. Of course, several caveats should be kept in mind whileth interpreting our results, namely: survival proxies did not include breeding success or the active period. Also, animals were confined to outdoor enclosures, which underestimates mortality due to predators. Finally, and obviously, sample sizes were limited, which reduces the statistical power to estimate other selection coefficients (e.g., quadratic). Further studies are warranted for the flexibilization of these limitations, to estimate the impact of *Dromiciops* populations into the energy turnover of the temperate rainforest ecosystem.

## Data Availability

All data will be available upon request to the corresponding author.
